# Similar incidence of DNA damage response pathway alterations between clinically localized and metastatic prostate cancer

**DOI:** 10.1186/s12894-019-0453-9

**Published:** 2019-05-06

**Authors:** Isaac E. Kim, Sinae Kim, Arnav Srivastava, Biren Saraiya, Tina Mayer, Wun-Jae Kim, Isaac Yi Kim

**Affiliations:** 1 The Warren Alpert Medical School of Brown University, Providence, RI USA; 2 Department of Biostatistics, Rutgers School of Public Health, The State University of New Jersey, New Brunswick, NJ USA; 3 Section of Urologic Oncology, Division of Urology, Rutgers Cancer Institute of New Jersey and Rutgers Robert Wood Johnson Medical School, Rutgers, The State University of New Jersey, 195 Little Albany Street, #4565, New Brunswick, NJ 08903 USA; 4 Department of Internal Medicine, Rutgers Robert Wood Johnson Medical School, Rutgers, The State University of New Jersey, New Brunswick, NJ USA; 5 Department of Urology, Chungbuk National University College of Medicine, Cheongju, South Korea

## Abstract

**Background:**

In this era of precision medicine, the DNA damage response (DDR) pathway has been shown to be a viable target of intervention in metastatic castration-resistant prostate cancer (CRPC) as approximately one-third of CRPC patients harbor DDR pathway mutations. To determine whether DDR pathway is a potential therapeutic target in localized disease, we analyzed The Cancer Genome Atlas (TCGA) in the present study.

**Methods:**

TCGA is a publically available cancer genome database that is sponsored by the United States National Cancer Institute. Total of 455 cases were available in the database at the time of this analysis.

**Results:**

DDR pathway gene mutations or copy number alterations were present in 136 (29.9%) of the 455 cases. On a univariate analysis, DDR pathway status did not correlate with serum prostate specific antigen, tumor stage or grade. However, among patients with high-risk features post-operatively (pathologic stage ≥ T3, Gleason score ≥ 8, or PSA > 20 ng/ml), DDR pathway alteration was associated with a lower overall survival (*p* = 0.0291).

**Conclusions:**

Collectively these results suggest that DDR pathway alterations may also be significant in localized prostate cancer and agents such as PARP inhibitors should be considered in patients with a high-risk disease.

## Background

Prostate cancer (PCa) remains the most common malignancy among men in the United States [1]. Due to the heterogeneity of the disease, recent studies have utilized next-generation sequencing to identify predictive biomarkers and provide molecular stratification. Identifying driver mutations which contribute to tumorigenesis and disease progression can lead to development and implementation of targeted therapy. High rates of genomic mutations in DNA damage repair (DDR) genes, such as breast cancer 2, early onset (BRCA2) and ataxia telangiectasia mutated (ATM) genes, have been detected in multiple malignancies [2–4]. More recently, it has been suggested that tumors with such homologous recombination defects may be sensitive to poly (adenosine diphosphate-ribose) polymerase (PARP) 1 inhibitors, such as olaparib [5, 6].

With regards to PCa specifically, approximately 30% of metastatic castration-resistant prostate cancer (CRPC) have been reported to contain an aberrant DDR pathway [7]. However, the full extent and prevalence of DDR pathway alterations has not been extensively analyzed in localized disease. Thus far two prior studies suggested the incidence to range 8–20% [8, 9]. Therefore, in the present study, we analyzed the largest publically available version of The Cancer Genome Atlas (TCGA) to assess the rate of altered DNA damage repair machinery in localized prostate cancer. We report that the incidence of DDR pathway alterations is significantly higher than previously thought and approaches 30%.

## Methods

Both the Provisional and Cell 2015 TCGA data were initially extracted as TSV files. At the time of this analysis, there were 455 patients in the Provisional TCGA database (http://www.cbioportal.org/index.do?session_id=5b8fc998498eb8b3d567b2ac). Three sets of data were extracted and compiled based on the following criteria: pathologic Gleason score and stage, pre-operative PSA, and survival. The Provisional data was used for the pathologic Gleason score and stage, the Cell 2015 data was used for pre-operative PSA, and both the Provisional and Cell 2015 data were merged for the survival.

The programming language Python was used to extract the samples in which the Neoplasm American Joint Committee on Cancer Clinical Distant Metastasis M Stage was M0. These samples were first separated into two categories, the first being normal (unaffected) and the second being altered (affected; include mutations and copy number alterations). Within these categories, the samples were then placed into sets based on three factors:

1. Pathologic Gleason score, divided into less than or equal to 6, equal to 7, and greater than or equal to 8, which was computed by summing the Gleason pattern primary and the Gleason pattern secondary.
2. Pathologic stage, divided into organ confined (T2) vs non-organ confined (≥ T3a).
3. Pre-operative PSA, divided into ranges [0–10], [10–20], and greater than or equal to 20, which was extracted by cross-referencing the given sample ID from the Provisional data to the Cell 2015 data.

The attributes that were recorded and placed into the respective Excel files that included the sample ID, the months that the patient was disease free, months of overall survival, and overall survival status.

Baseline patient and clinical characteristics were summarized using descriptive statistics. Fisher’s exact test was performed to test the association between two categorical variables. Student’s t-test was used to compare mean age between the affected and unaffected group. The association of DDR mutation status with time from diagnosis to death from any cause was evaluated using log-rank tests and Kaplan-Meier curve estimates were plotted. All statistical tests were two-sided and *p* < 0.05 was considered to indicate statistical significance. All analyses were performed by Python and SAS 9.4 (Cary, NC).

## Results

**The overall incidence of DDR pathway alterations in localized prostate cancer approaches 30%.** To assess the potential pathologic significance of DDR pathway in localized prostate cancer, we analyzed the TCGA prostate cancer database for mutations or copy number alterations of the following twelve genes (CHEK1, CHEK2, RAD51, BRCA1, BRCA2, MLH1, MSH2, ATM, ATR, MDC1, PARP1, and FANCF). The results revealed that 136 of the 455 cases were affected (29.9%), with 54 containing mutations and 92 copy number alterations (CNAs) (11.9 and 20.2%, respectively). The summary statistics of the patients with DDR pathway alterations are shown in Table 1. In men with DDR pathway alterations, an organ confined (pT2) and non-organ confined disease (pT3/4) comprised 34.6 and 65.4%, respectively (*p* = 0.57). With respect to pathologic Gleason score 6, 7, and ≥ 8, the proportion affected was 6.6, 49.3, and 44.1% (*p* = 0.61).

**Table 1:** Summary statistics on DNA Damage Response pathway alterations in clinically localized prostate cancer

Parameters	Normal (unaffected)	Mutated (affected)	*p*-value
Total sample size, *N*	319	136	
Age (years), median (range)	61 (41–77)	62 (43–78)	0.034
PSA pre-operative, median (ng/ml), (range), *n*	6.8 (2.2–56.4), 125	9.7 (2.2–87.0), 39	Not significant
Stage, pathologic American Joint Committee on Cancer Tumor Stage Code, *N* (%)			Not significant
pT2	127 (39.8)	47 (34.6)	
pT3	186 (58.3)	86 (63.2)	
pT4	6 (1.9)	3 (2.2)	
Gleason score, pathologic, *N* (%)			Not significant
6	28 (8.8)	9 (6.6)	
7	163 (51.1)	67 (49.3)	
8–10	128 (40.1)	60 (44.1)	

**Fig. 1:**
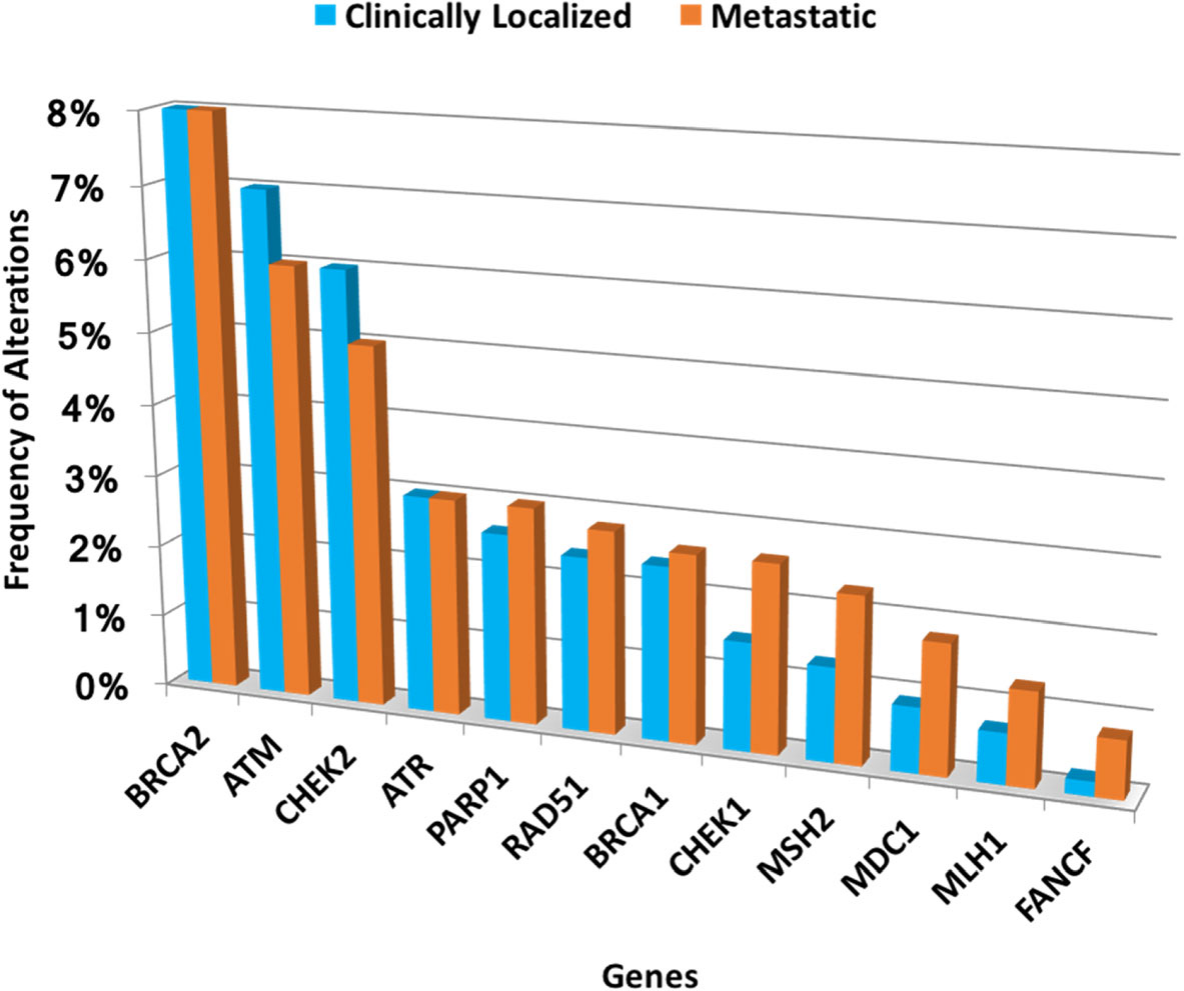
Breakdown of DDR pathway alterations in localized and metastatic prostate cancer. Most frequent DDR pathway alterations were BRCA2 and ATM for both localized and metastatic prostate cancer

**Fig. 2:**
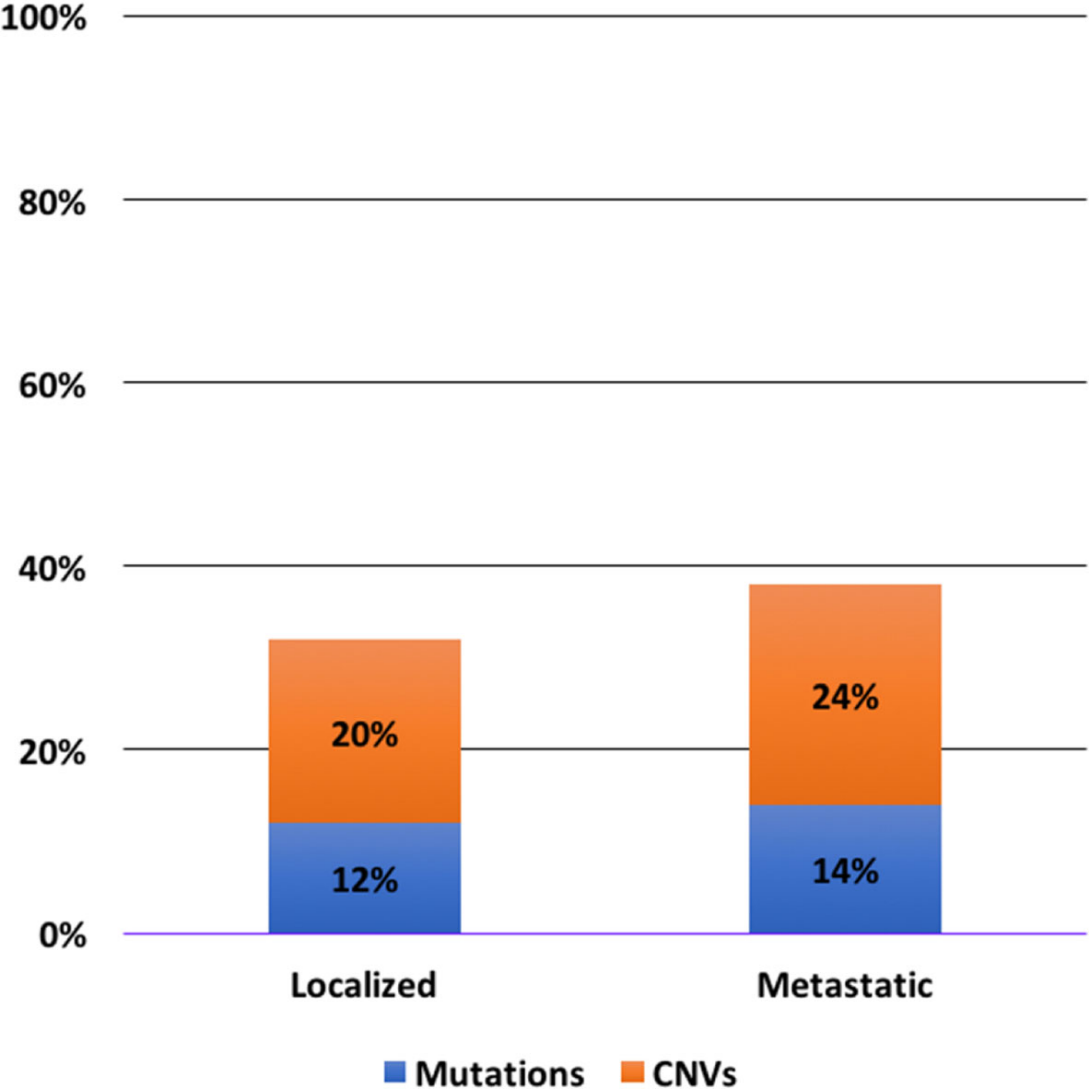
Breakdown of mutations and copy number alterations in DDR pathways. The overall proportion of mutations and copy number alterations were similar for localized and metastatic prostate cancer

Figure 1 illustrates the breakdown of the affected genes from the data base analysis. As a comparison, similar analysis was carried out for the metastatic castration resistant prostate cancer (mCRPC) cases by pooling three databases within the cbioportal website [10–12]. The overall rate of DDR pathway alteration in this pooled dataset was 33% (91 of 272 patients). The proportion of altered cases between mutations and CNAs was similar to that of the localized cases (14 and 24%, respectively) (Fig. 2).

**Fig. 3:**
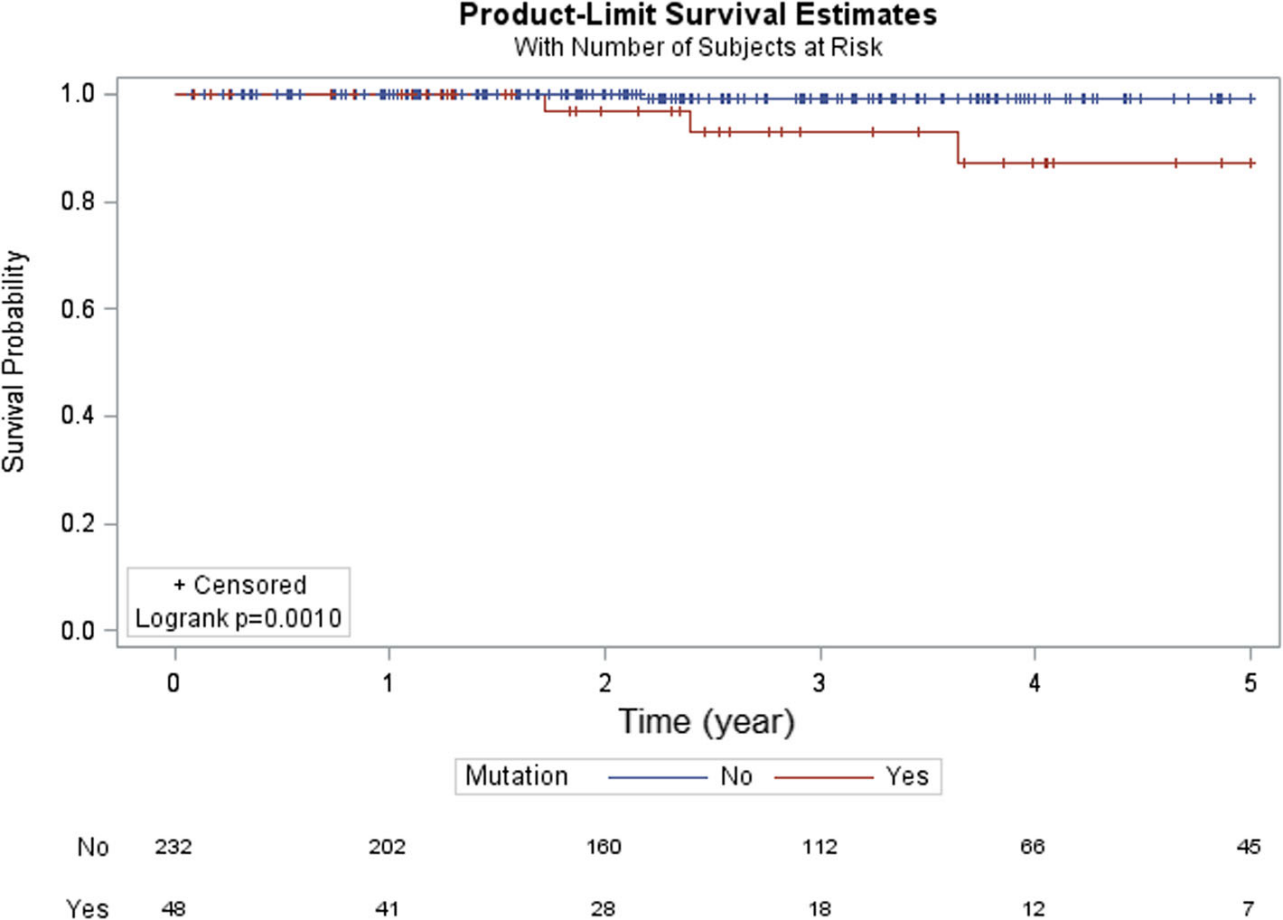
Overall survival in pT3 or higher stage prostate cancer. DDR pathway alteration was associated with a shorter overall survival in patients with pT3 or higher stage prostate cancer

**Fig. 4:**
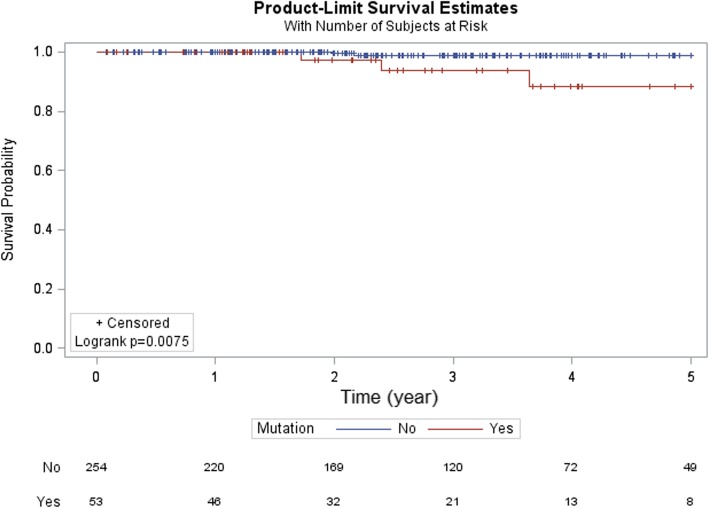
Overall survival in high risk prostate cancer. In men with high risk prostate cancer (pT3 or higher, Gleason score 8 or higher, or pre-operative PSA greater than or equal to 20), DDR pathway alteration was associated with a shorter overall survival

**DDR pathway alterations associated with poor prognosis in men with high-risk prostate cancer.** Next, we stratified the data based on pre-operative PSA and pathologic Gleason score and stage and investigated the potential prognostic value of DDR pathway alterations. Of the 455 cases in the database, survival data was available in 371 while pre-operative PSA in 164. The results were not significant for pre-operative PSA and pathologic Gleason score. However, in patients with pT3 or higher disease, DDR pathway alterations was associated with a lower overall survival (OS) (Fig. 3, *p* = 0.0046). In addition, in men with features of high-risk of recurrence following radical prostatectomy (pathologic Gleason score 8 or higher, pathologic stage T3 or higher, or pre-operative PSA ≥ 20 ng/ml), DDR pathway alterations again correlated with a shorter OS (Fig. 4, *p* = 0.0291).

## Discussion

In the present study, we investigated DDR pathway alterations in localized prostate cancer using the TCGA database. We found that DDR pathway alteration rate is surprisingly high and occurred in approximately 30% of the cases. In addition, DDR pathway alteration was associated with a shorter OS in men with high-risk features post-operatively. These results suggest that a dysregulated DDR pathway may occur earlier during prostate cancer progression than previously thought and that available inhibitors of DDR pathway may have a therapeutic role in localized prostate cancer.

The observed DDR pathway alterations rate in localized prostate cancer was significantly higher than expected based on the reported range of 8–20% [8, 9]. Previously, DDR pathway has been reported to be altered in the 30% range in men with mCRPC. For example, in the landmark clinical trial that investigated the role of the PARP inhibitor olaparib [7], DDR pathway mutations were seen in 33% of mCRPC. Consistent with such result, we have also observed that the percentage of metastatic prostate cancer patients with DDR pathway mutations or copy number alterations was 33% in three publically available databases. In this context, the present observation that 30% of the TCGA’s prostate cancer cases contain DDR pathway alterations is significantly higher than expected because the database represents entirely a non-metastatic, clinically localized prostate cancer. Accordingly, alterations in DDR pathway may occur earlier during prostate carcinogenesis.

The present finding also has therapeutic implications. Currently, there are multiple agents such as olaparib, niraparib, and rucaparib [5, 13, 14] that target the DDR pathway. Among these PARP inhibitors, olaparib has been shown to be effective in men with mCRPC as a monotherapy and in combination with abiraterone [7, 15]. Since DDR pathway alterations were seen at similar rate between localized and metastatic prostate cancer, it is plausible that PARP inhibitors may also have a therapeutic effect in localized prostate cancer. To test this possibility we are currently designing an adjuvant trial that will assess the effect of a PARP inhibitor in prostate cancer patients with high-risk features post-operatively.

It should be noted that a recent manuscript also investigated the DDR pathway in localized prostate cancer using the same TCGA database [9]. However, there were two significant differences between the present and the aforementioned study. First, the sample size analyzed is significantly larger with the current study as TCGA provisional data was used as compared to the TCGA Cell 2015 [8]. Second, as indicators of pathway alterations, we included both mutations and CNAs while the published study limited the investigation to mutations. Although the precise biological differences between mutations and CNAs is not clear, CNAs likely also represent a dysregulated pathway and should be analyzed in any therapies that target the DDR pathway.

This study has limitations. First, TCGA database does not contain all the relevant clinical information. Second, the biological significance of DDR pathway alterations cannot be assessed. Indeed, as indicators of altered DDR pathway, both mutations and CNAs were included. It is entirely possible that these various types of genetic changes may have different pathologic implications. Nevertheless, the present results suggest that DDR pathway alterations in localized prostate cancer is similar to that of heavily treated mCRPC, which supports further investigations into therapeutic strategies in this population.

## Conclusions

DDR pathway alterations in localized prostate cancer in the TCGA database was approximately 30%. Such high rate suggests that agents such as PARP inhibitors may be an effective part of the treatment armamentarium in localized prostate cancer.

## Abbreviations

CRPC: Castration-resistant prostate cancer
DDR: DNA Damage Response
mCRPC: Metastatic castration-resistant prostate cancer
OS: Overall survival
PARP: Poly (adenosine diphosphate-ribose) polymerase
PCa: Prostate cancer
TCGA: The Cancer Genome Atlas
